# Single-crystalline FeCo nanoparticle-filled carbon nanotubes: synthesis, structural characterization and magnetic properties

**DOI:** 10.3762/bjnano.9.95

**Published:** 2018-03-29

**Authors:** Rasha Ghunaim, Maik Scholz, Christine Damm, Bernd Rellinghaus, Rüdiger Klingeler, Bernd Büchner, Michael Mertig, Silke Hampel

**Affiliations:** 1 Leibniz Institute for Solid State and Material Research Dresden, Helmholtzstrasse. 20, 01069 Dresden, Germany; 2 Institute for Physical Chemistry, Technische Universitaet Dresden, 01062 Dresden, Germany; 3 Kirchhoff Institute for Physics, Heidelberg University, Im Neuenheimer Feld 227, D-69120 Heidelberg, Germany; 4 Center for Advanced Materials (CAM), Heidelberg University, Im Neuenheimer Feld 225, D-69120 Heidelberg, Germany; 5 Institute for Solid State Physics, Technische Universitaet Dresden, 01062 Dresden, Germany; 6 Kurt-Schwabe-Institut für Mess- und Sensortechnik e.V. Meinsberg, 04736 Waldheim, Germany

**Keywords:** carbon nanotubes, crystal structure, encapsulation, Fe–Co binary nanoparticles, magnetic nanoparticles

## Abstract

In the present work, we demonstrate different synthesis procedures for filling carbon nanotubes (CNTs) with equimolar binary nanoparticles of the type Fe–Co. The CNTs act as templates for the encapsulation of magnetic nanoparticles and provide a protective shield against oxidation as well as prevent nanoparticle agglomeration. By variation of the reaction parameters, we were able to tailor the sample purity, degree of filling, the composition and size of the filling particles, and therefore, the magnetic properties. The samples were analyzed by scanning electron microscopy (SEM), transmission electron microscopy (TEM), X-ray diffraction (XRD), superconducting quantum interference device (SQUID) and thermogravimetric analysis (TGA). The Fe–Co-filled CNTs show significant enhancement in the coercive field as compared to the corresponding bulk material, which make them excellent candidates for several applications such as magnetic storage devices.

## Introduction

Research on nanoscale materials is motivated by the observation that properties of materials may completely change when a bulk material is scaled down to its smallest size [1–4]. One of the driving forces is the greatly enhanced surface-to-volume (S/V) ratio of nanomaterials. At the same time, this ratio constitutes a major challenge, as a large surface area can trigger rapid oxidation, especially in metallic nanoscale objects [5].

The possibility to fabricate nanomaterials and nanocomposites opens the door to numerous applications in multidisciplinary fields, such as magnetic storage [6–7], fuel cells [8], electromagnetic wave absorption [9], sensors for magnetic force microscopy [10] and human tumor therapy [11–13]. Fe–Co binary alloys are of particular interest due to their high saturation magnetization, large permeability and high magnetophoretic mobility [14], which make them suitable as magnetic carriers for bioseparation and drug delivery [15–16]. Fe–Co bulk alloys are soft magnetic materials which have the largest known saturation magnetization per atom (2.45 µB/atom) [17–18] in addition to low coercivity and a high Curie temperature (*T*_c_ ≈ 900 °C) [19–20], and are thus highly suitable for high-temperature applications. However, reducing the size of these materials down to the nanoscale to produce nanowires and nanoparticles results in a robust enhancement in the coercivity [21–22], which make them good candidates for high-density magnetic storage devices.

Different techniques have been applied for the synthesis of these magnetic nanoparticles (MNPs), such as mechanical alloying [23], electrodeposition [24], radio frequency (rf)-plasma torch [25], sol–gel methods [26–27], reverse micelle systems [28] and thermal decomposition of bimetallic alloys [29]. However, many of these techniques are relatively expensive (either high-cost starting materials or high-energy consumption), and involve difficulties in controlling the size and morphology of the nanoparticles. Since there is a clear correlation between the morphology, size, size distribution, shape, arrangement of the magnetic nanoparticles to their magnetic, chemical, mechanical and catalytic properties [18,26,30], it is important to choose synthesis methods which guarantee the control of size and morphology as well as the long-term stability in order to allow these magnetic nanoparticles to be used in potential applications.

Due to their high surface-to-volume (S/V) ratio, MNPs are more susceptible to oxidation, agglomeration, aggregation [31–32]. Therefore, it is necessary to produce MNPs with a protective layer which preserves their properties. Compared to polymer or silica coatings, carbon nanotubes (CNTs) have been introduced as a protective shell due to their high stability in different chemical and physical environments such as acidic, basic, high temperature and pressurized conditions [33–36]. CNTs can also act as a template to control the size and morphology of the filling material due to the confinement of the material within the hollow tubular cavity. Chemical vapor deposition (CVD) is a technique used to fill MNPs into CNTs via in situ filling, in which metallocene precursors are used as a carbon source and MNPs [22,30,37] or hydrocarbons (such as benzene) can be used as carbon precursors, which can be decomposed in an inert atmosphere over freshly prepared alloys [33]. However, post-synthesis filling is a facile method for filling the CNTs via wet chemistry. CNTs can act as molecular straws that are able to suck material into the tubular cavity via capillary force [38–40].

Due to the attractive properties of Fe–Co nanoparticles, this work is directed towards the synthesis of CNT-based nanocomposites of Fe–Co based on a post-synthesis method, in which prefabricated multiwalled CNTs (MWCNTs) are used as templates. Two facile filling approaches have been applied, and a study on the morphology, structure and magnetic properties of the resulting Fe–Co MNPs is presented. The promising effect of an additional heat treatment step on these properties is also investigated.

## Experimental

### Preparation of the binary alloys inside CNTs

Multiwalled carbon nanotubes (MWCNTs) of the type PR-24-XT-HHT (Pyrograf Products, Inc., Cedarville OH, USA) have been used as templates or nanocontainers for the preparation of intermetallic nanoparticles. This type of CNT is distinguished by its high purity which results from the fact that the as-produced carbon nanotubes are heat treated to 3000 °C. This process reduces the iron content (i.e., the catalyst) to a very low level (<100 ppm) [41–43]. The material Fe_50_Co_50_@CNT has been prepared by the following two filling approaches.

The first approach is an extension of a reported solution filling approach for CNTs [44–45]. 1 M standard aqueous solutions of the following nitrates have been prepared: Fe(NO_3_)_3_·9H_2_O (grade: ACS 99.0–100.2%) and Co(NO_3_)_2_·6H_2_O (grade: ACS 98.0–102.0% metal basis) supplied by VWR Chemicals and Alfa Aesar GmbH & Co KG (Karlsruhe, Germany). The nitrate salts were used as provided and no further purification was performed. The solutions were combined in a stoichiometric ratio with respect to the metal ions (i.e., Fe/Co 1:1), where about 100 mg of MWNTs were added and the mixture was treated in an ultrasonic bath for 45 min at room temperature. The mixture was then vacuum-filtered and washed with about 20 mL of washing agent (acetone and distilled water of a volumetric ratio of 1:1). The solid residue was then dried for 24 h at a temperature of 108 °C and reduced under hydrogen and argon atmosphere (50 vol % Ar + 50 vol % H_2_) at a temperature of 500 °C for 4 h to convert all the nitrates and oxides to the corresponding metallic state [34,46]. An additional heat treatment step was essential to obtain the desired intermetallic phase in which the reduced sample was further annealed under a mixture of Ar and H_2_ gases streams (95 vol % Ar + 5 vol % H_2_) at a temperature of 600 °C for 48 h.

In an attempt to obtain a relatively higher degree of filling, a second approach was followed in which the nitrate precursors were directly mixed with the specific amount of CNTs (mainly 50 mg) in a sealed round bottom flask. A few drops of distilled water were added to ensure good stirring. The flask with the mixture was placed in an oil bath and heated to a temperature of around *T* ≈ 65 °C for 4 h. The mixture was then naturally cooled down to room temperature and washed with about 20 mL washing agent of acetone and distilled water of a volumetric ratio of 1:1. The samples were then dried, reduced and annealed in a manner similar to the solution filled samples. This approach differs from the solution method in that the liquid medium of the filling material is provided with a higher saturation (due to the absence of water solvent), which increases the percentage of material that fills the CNTs.

### Characterization

All samples were routinely investigated by scanning electron microscopy (SEM) with a Nova 200 NanoSEM from FEI Company operated at 15 kV and combined with energy dispersive X-ray (EDX) analyzer (AMETEK). The SEM samples were prepared by placing a thin film of the sample on carbon tape. Transmission electron microscopy (TEM), high-resolution transmission electron microscopy (HRTEM) measurements and nanobeam electron diffraction patterns were performed using a Tecnai F30 (FEI) instrument operated at 300 kV or a Tecnai G2 (FEI) instrument operated at 200 kV. Both were equipped with an EDX analyzer (AMETEK, Oxford). The TEM samples were prepared by adding a few drops of the sample suspension in acetone on a copper grid with a carbon coating on one side.

The crystal structure of the magnetic nanoparticles inside the CNT was identified using an X’Pert Pro MPD PW3040/60 X-ray diffractometer (XRD) (Panlytical) with Co Kα radiation (λ = 1.79278 Å) in reflection geometry at a scanning rate of 0.05° s^−1^ in the 2θ range from 10° to 80°.

Thermogravimetric measurements (TGA) were performed with a SDT-Q600 (TA Instruments) instrument. A few milligrams of the material (≈5 mg) were heated to a temperature of 900 °C with a heating rate of 5 K/min followed by an isothermal of 15 min under air atmosphere with a flow rate of 100 mL/min.

The magnetic field dependence of the magnetization at 5 K and 300 K in an external magnetic field up to ±5 T was measured by means of superconducting quantum interference device (MPMS-XL SQUID) magnetometer from Quantum Design (San Diego CA, USA). The samples were filled inside gelatin capsules, and the diamagnetic contribution of the sample holder and the empty CNT was subtracted.

## Results and Discussion

### Morphology and structure

**Figure 1:**
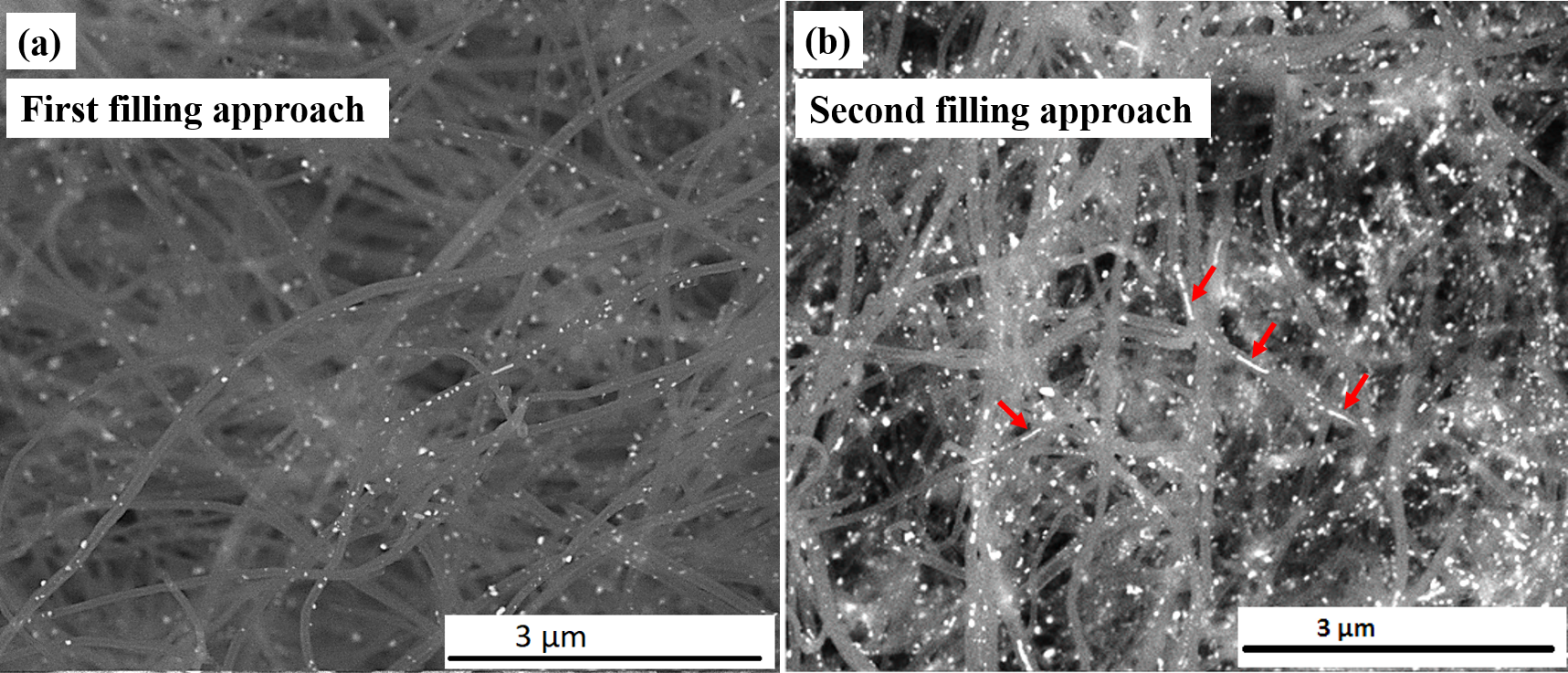
SEM overview images in back scattering electron (BSE) contrast mode for the as-prepared samples of Fe_50_Co_50_@CNT prepared by the a) first (solution) and b) second filling approach.

The morphology and geometry of the filling material and its location inside or outside the CNTs was examined by SEM (Figure 1).

Figure 1a shows an overview image in back scattered electron (BSE) mode for the as-prepared sample of Fe_50_Co_50_@CNT prepared by the first (i.e., solution) filling approach. The filling particles are distributed along the inner cavity of the hollow CNTs. For samples prepared by the second filling approach (Figure 1b), the same behavior is observed, however with a seemingly higher degree of filling compared to the solution approach. This can be seen from the pearl necklace-like appearance of the filling inside the CNTs and was confirmed by quantitative measurements performed by TGA as will be shown later (see Figure 6).

**Figure 2:**
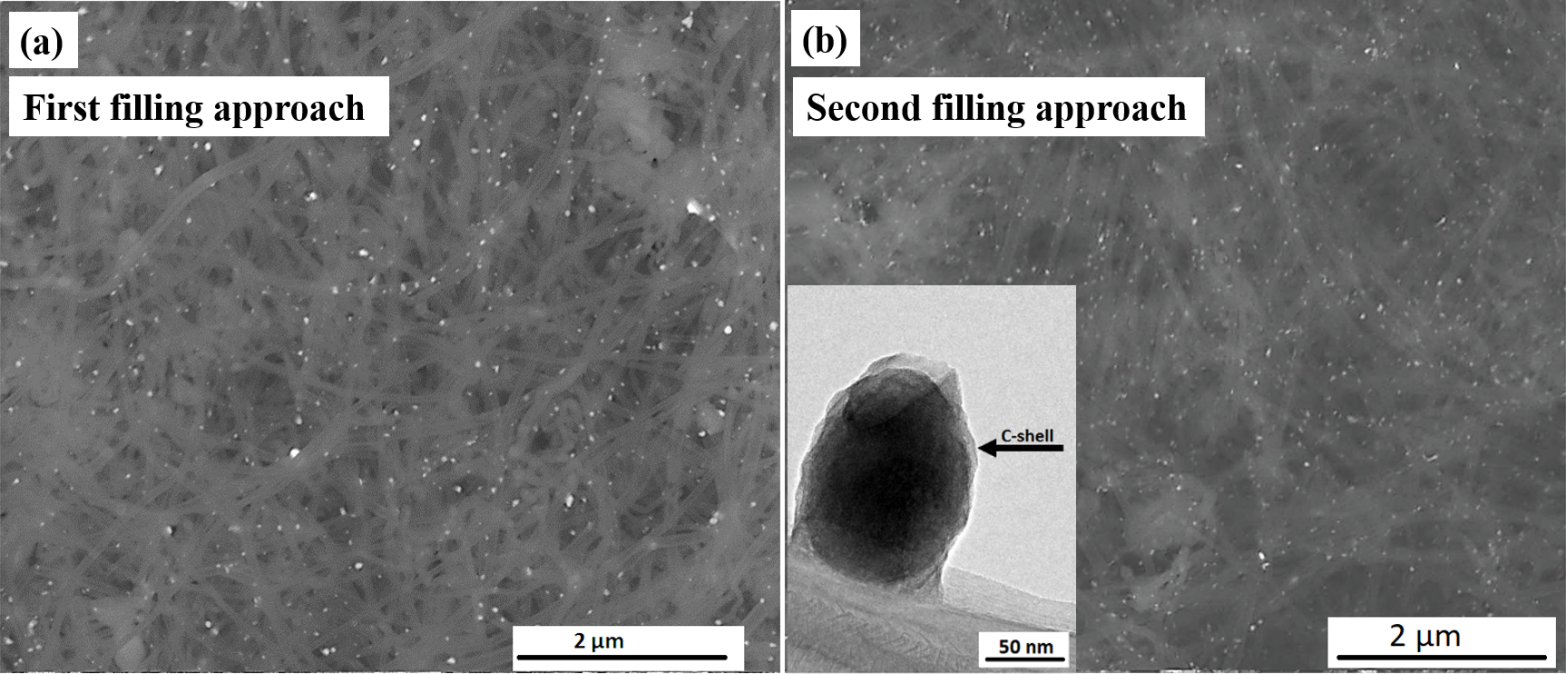
SEM overview images in BSE contrast of the annealed samples of Fe_50_Co_50_@CNT (at 600 °C for 48 h) prepared by the a) first (solution) and b) second filling approach. Inset: a MNP, attached to the outer surface of a CNT, and covered with a carbon shell.

It is important to emphasize the effect of annealing on the growth of the particles. For the as-prepared samples (i.e., only reduced, Figure 1a and 1b), different morphologies and particle sizes for the filling materials have been observed (small, large spheres, particle chains as indicated by arrows in Figure 1b), whereas after an additional heat treatment step (annealing at 600 °C for 48 h), a significant growth in the particles size was observed. This observation was confirmed by statistical measurements of the particle aspect ratio and the diameter with respect to the diameter of the inner wall of the CNT. Figure 2a and Figure 2b show the corresponding BSE images for samples prepared by the first and second filling approaches, respectively, after an additional heat treatment step. We attribute the observation of a pronounced increase in particle size to the prolonged heat treatment (i.e., annealing for 48 h), which provides a significantly higher amount of thermal energy. This increases the probability of particle merging, which in turn leads to particle growth.

**Figure 3:**
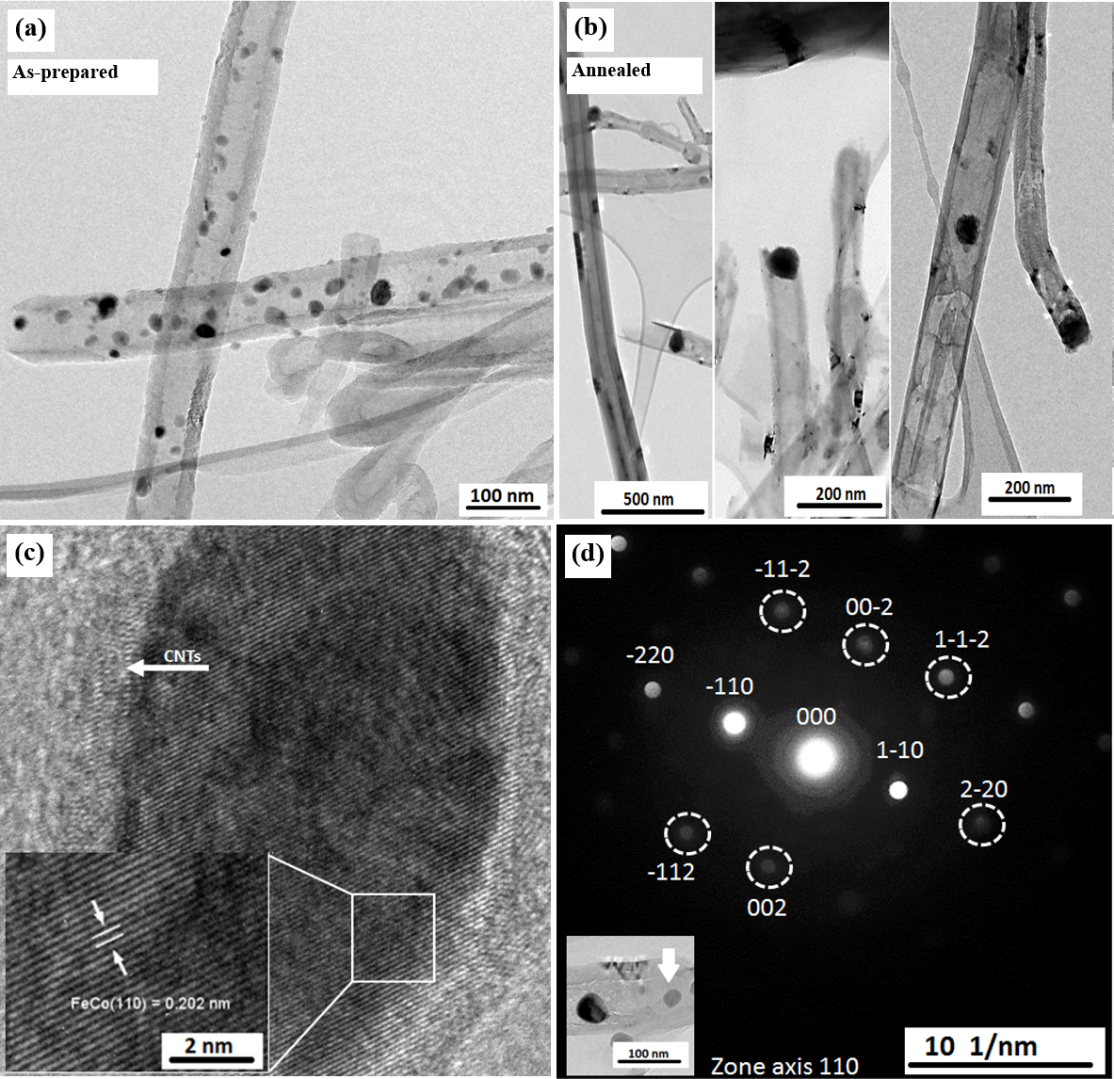
TEM bright field images for the a) as-prepared and b) annealed samples of Fe_50_Co_50_@CNT prepared by the second filling approach. c) HRTEM images for the as-prepared sample with the corresponding lattice fringes detailed in the inset, and d) nanobeam electron diffraction patterns of Fe_50_Co_50_ nanoparticles with the corresponding TEM image as inset.

TEM measurements were performed for samples prepared by both filling approaches, for each of the as-prepared and annealed samples. Figure 3a is an example of an as-prepared sample of Fe_50_Co_50_@CNT prepared by the second filling approach, in which most of the particles are located within the hollow cavity of the CNTs and exhibit a broad variety of sizes. All particles have a diameter much lower than the inner diameter of the CNTs, whereas after further heat treatment (Figure 3b), most of the particles reached a diameter which nearly equals to the diameter of CNT inner walls.

The morphology of the individual nanoparticles of Fe_50_Co_50_@CNT was investigated by HRTEM measurements. Surprisingly, some particles in the as-prepared sample showed high crystallinity, even without annealing, as shown in Figure 3c. The crystallinity of the core material was confirmed by the appearance of the lattice fringes (marked by short white parallel lines in Figure 3c), in which the bcc structure of Fe–Co can be identified from the interplanar distance of 0.202 nm (110). However, the need to anneal these samples arose from the magnetic property measurements (see Figure 7). TEM-based nanobeam electron diffraction carried out on several individual nanoparticles (≈15–20 nanoparticles in each investigated sample) for the annealed samples revealed that the filling particles are single crystalline, as indicated by the reflections corresponding to the 110 (0.202 nm), 211 (0.117 nm), 220 (0.102 nm) and 222 (0.085 nm) lattice planes confirming the bcc structure of Fe–Co phase (Figure 3d).

**Figure 4:**
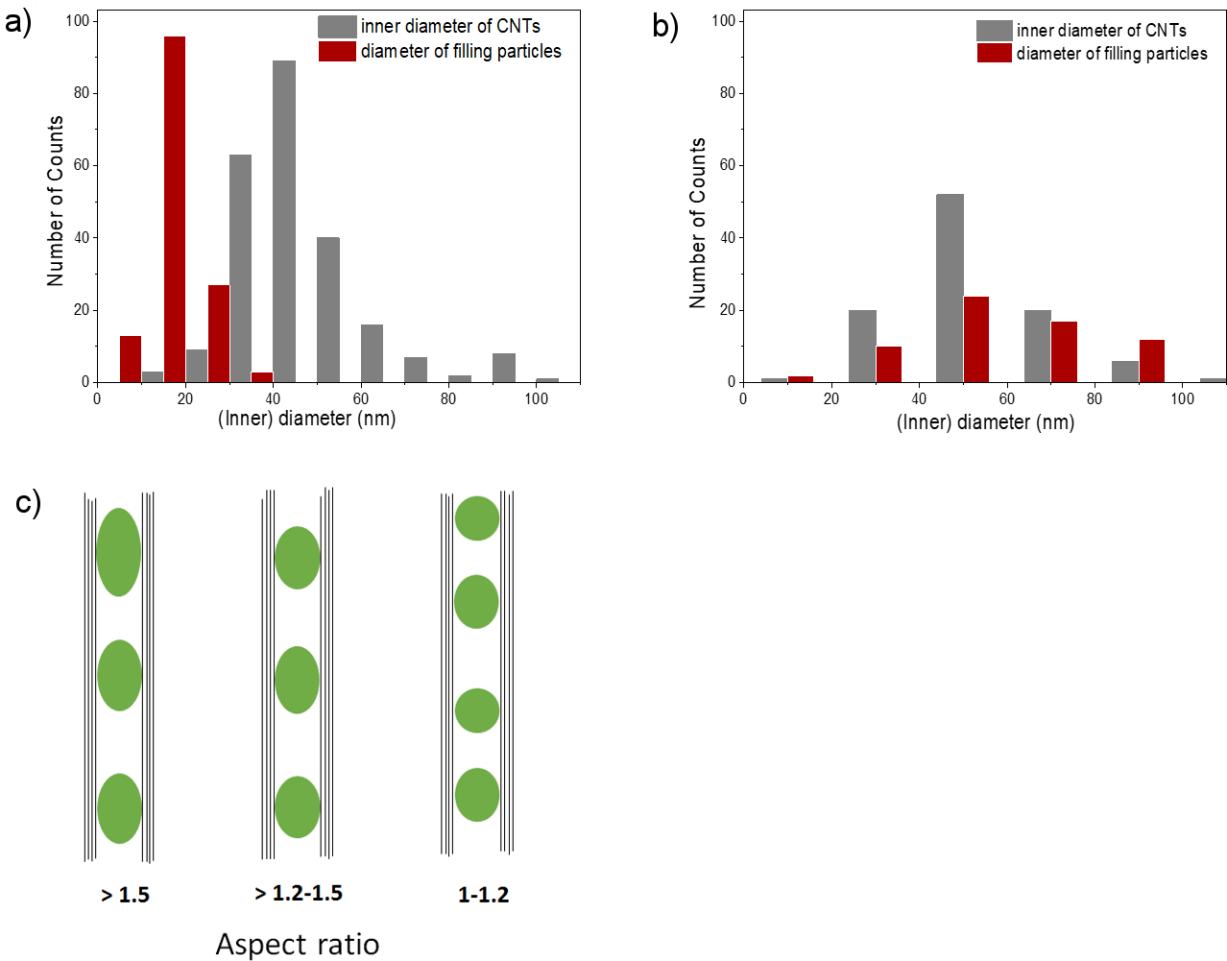
Histograms representing the distribution of the inner diameter (nm) of the CNTs and particle diameter for the a) as-prepared and b) annealed samples prepared by the second approach. c) Schematic representation of the geometry of the filling particles with respect to the aspect ratio values.

The distribution of the particle diameter was investigated for the as-prepared and annealed samples prepared by the second approach. The diameters were measured perpendicular to the long axis of the CNTs. The as-prepared samples have a mean diameter of *d*_TEM_ = 16 ± 5 nm (Figure 4a), whereas the annealed samples have a mean diameter of *d*_TEM_ = 58 ± 20 nm (Figure 4b). Comparing these values with the mean diameter of the hollow cavity of the CNTs (*d*_CNT_ = 53 ± 20 nm), one can conclude that confinement of the magnetic nanoparticles to the inner diameter of the CNTs allowed the control of the particle size, and to a large extent, prevented particle agglomeration in both samples. Furthermore, a strong divergence between the size distribution of the as-prepared and the annealed samples is obvious. Hence, for very regular particles with high homogeneity and crystallinity, an annealing step is necessary.

The rather spherical geometry of the intermetallic nanoparticles inside CNTs was confirmed by TEM aspect ratio studies (i.e., the ratio of the particle’s long axis to its short axis).

In the as-prepared sample (with nearly 140 investigated filling particles), 57% of the particles had aspect ratios of about 1–1.2, whereas 33% of the particles had aspect ratios in the range of 1.2–1.5. The remaining 10% exhibited ratios larger than 1.5. However, in an annealed sample (with nearly 60 investigated filling particles), 76% of the particles had aspect ratios of about 1–1.2, whereas 24% had aspect ratios larger than 1.2, but did not exceed 1.5. Hence, the morphology became more homogenous after further heat treatment. We attribute the homogeneity in the morphology of the annealed samples to the non-wetting behavior of the Fe–Co alloy nanoparticles and carbon nanotubes [39], in which the contact area between the nanoparticles and the tubes tends to minimize. Hence, when annealing takes place, the filling particles tend to agglomerate and form spherical particles. Figure 4c shows a schematic representation of the geometry of the filling particles based on the aspect ratio values.

The expected stoichiometry of 1:1 for the binary alloys was confirmed by EDX measurements, in which the EDX analyzer was attached to both the SEM and TEM. In SEM-EDX, the stoichiometry was obtained by measuring the relative ratio of the individual elements over an analyzed area of about 60 × 50 µm^2^ (see Figure S1 in Supporting Information File 1), whereas in TEM-EDX, the relative ratio was obtained for a large number of individual nanoparticles. Quantitative analysis indicates the average atomic percentage of Fe is 51.2 ± 1.0 atom % and 48.8 ± 1.0 atom % for Co in Fe_50_Co_50_@CNT samples prepared by both filling approaches.

**Figure 5:**
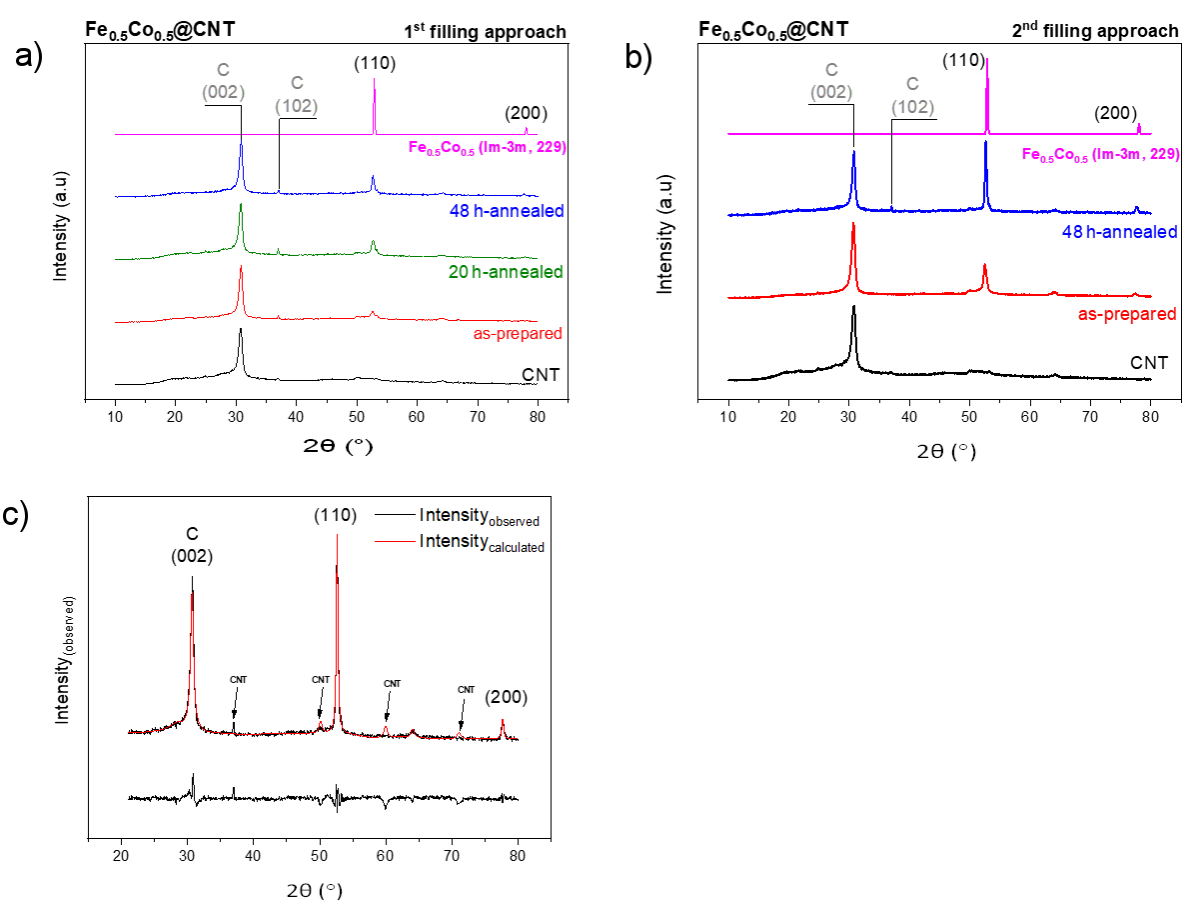
XRD diffraction patterns for the as-prepared and annealed samples of Fe_50_Co_50_@CNT prepared by the a) first (solution) and b) second approach. c) Rietveld refinement for the annealed Fe_5 0_Co_50_@CNT sample prepared by the second approach.

The XRD diffraction patterns for the as-prepared and annealed samples prepared by the first and second filling approaches are shown in Figure 5a and Figure 5b, respectively.

Two main characteristic features can be deduced from the diffraction patterns: (1) the intensity of the main peak (corresponding to the 110 plane) in the sample prepared by the second approach is larger than the corresponding one in the solution filling approach. This is mainly due to the higher degree of filling due to the absence of water during filling, which results in a higher order of crystallinity and thus a larger number of 110 planes. Higher filling is also confirmed by TGA data. (2) The reflection intensities in general increase with annealing, which means that more particles of the homogenous bcc phase of Fe_50_Co_50_ are formed. The intense reflections at 2θ = 30° and 37 ° correspond to the 002 and 102 lattice planes, respectively, of CNTs (labeled with C). The reflections at 2θ = 53° and 78° correspond to the lattice planes 110 and 200, respectively, for the bcc structured Fe_50_Co_50_ with space group Im-3m (229, cubic, PDF No. 04-003-5514) [22]. No reflections corresponding to oxides or carbides were detected. It is worth to mention that Zhang et al. reported the synthesis of Fe–Co nanoparticles encapsulated inside CNTs in a similar way to our first filling approach [45]. However, XRD diffraction patterns revealed the coexistence of several alloy phases with different Fe/Co ratios (e.g., Fe_3_Co_7_, FeCo, Fe_7_Co_3_), whereas our XRD diffraction pattern revealed the presence of a homogenous bcc phase of pure Fe_50_Co_50_ nanoparticles.

Rietveld refinement identifying the exact structure of an annealed sample of Fe_50_Co_50_@CNT prepared by the second filling approach was also carried out as displayed in Figure 5c. The intense reflection at 2θ = 30° corresponds to the 002 lattice plane of the CNTs. The reflections at 2θ = 53° and 78° correspond to the lattice planes 110 and 200, respectively, of the bcc structure Fe_50_Co_50_ with space group Im-3m. The reflections indicated by arrows refer to the CNTs, whereas here a graphite diffraction pattern was taken as a standard.

Assuming the spherical shape of the nanoparticles, the mean particle diameter was calculated using Scherrer’s equation, given by [47]:


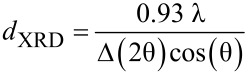


where *d*_XRD_ is the mean size of the particles, λ is the X-ray wavelength (Co Kα, λ = 1.7927 Å) and Δ(2θ) is the line broadening at half the maximum intensity (FWHM) in radians. Using this model, the mean particle diameter *d*_XRD_ for an annealed sample prepared by the second approach equals to 47 ± 1 nm, which correlates with the mean particle size obtained from the statistical analysis of the TEM measurements (*d*_TEM_ = 58 ± 20 nm, Figure 4b), indicating that the TEM images show single crystals. Note, however, that the error bars in *d*_XRD_ refer to the measurement error while for *d*_TEM_ it indicates the observed size distribution. The diameter for the as-prepared sample is *d*_XRD_ = 19 ± 4 nm and is also in accordance with the TEM results (*d*_TEM_ = 16 ± 5 nm, Figure 4a).

**Figure 6:**
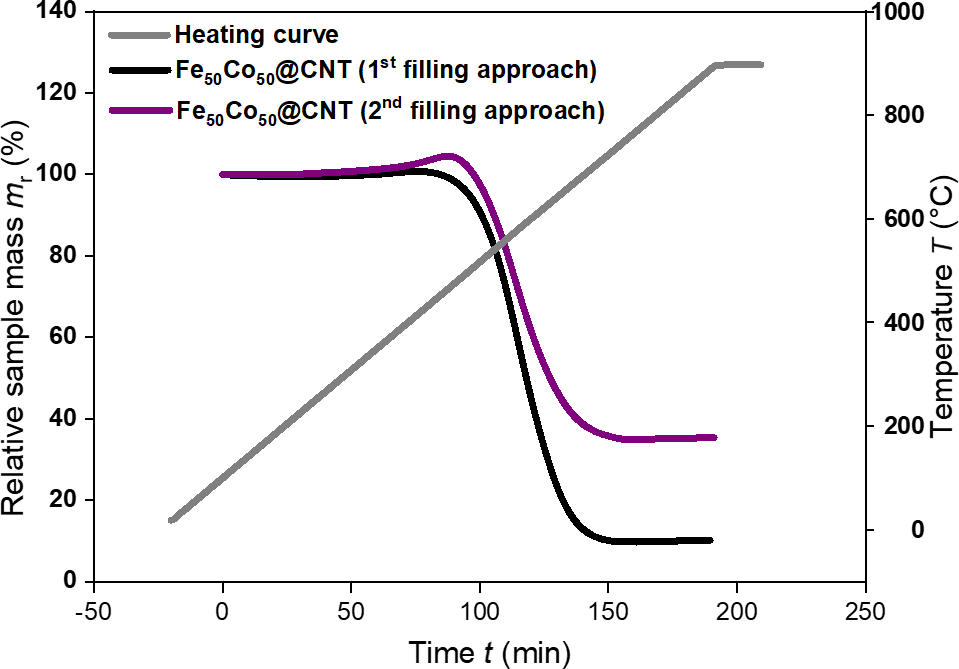
Relative sample mass loss for a sample prepared by the first (i.e., solution) approach (black) and the other by the second approach (purple) of Fe_50_Co_50_@CNT during the combustion process of the nanocomposite in which the CNT mass start to decrease at *T* ≈ 530 °C.

TGA is mainly used as a measure of the sample purity (i.e., the absence of outside particles which exhibit an increase in mass prior to the combustion of the CNTs), and for the determination of the filling material inside CNTs by performing back calculations based on the mass of the TGA residue [48–49]. This measurement confirms the observations found by SEM that the main difference between the two filling approaches is the filling yield, i.e., samples prepared by the second filling approach are found to have a higher filling yield in comparison with those prepared by the first (solution) filling approach. For some samples, the filling yield reached ≈8 ± 1 wt % for solution-filled samples (Figure 6, black curve), whereas for samples prepared by the second approach, the filling yield was as high as 20 ± 1 wt % (Figure 6, purple curve). The lower filling yield of the solution-filled samples can be attributed to the occupancy of the inner volume of CNTs by water, which decreases the filling capacity of the inner CNT cavity, compared to the available filling volume in the second filling approach, in which only a few drops of distilled water is required, added only to ensure good stirring. The humped peak observed for the samples filled by the second approach indicates the presence of particles attached to the outer surface of the CNTs, which are coated with carbon layers (see inset Figure 2b). Therefore, no diffraction peaks for the corresponding oxides were observed as shown by XRD measurements.

### Magnetic properties

**Figure 7:**
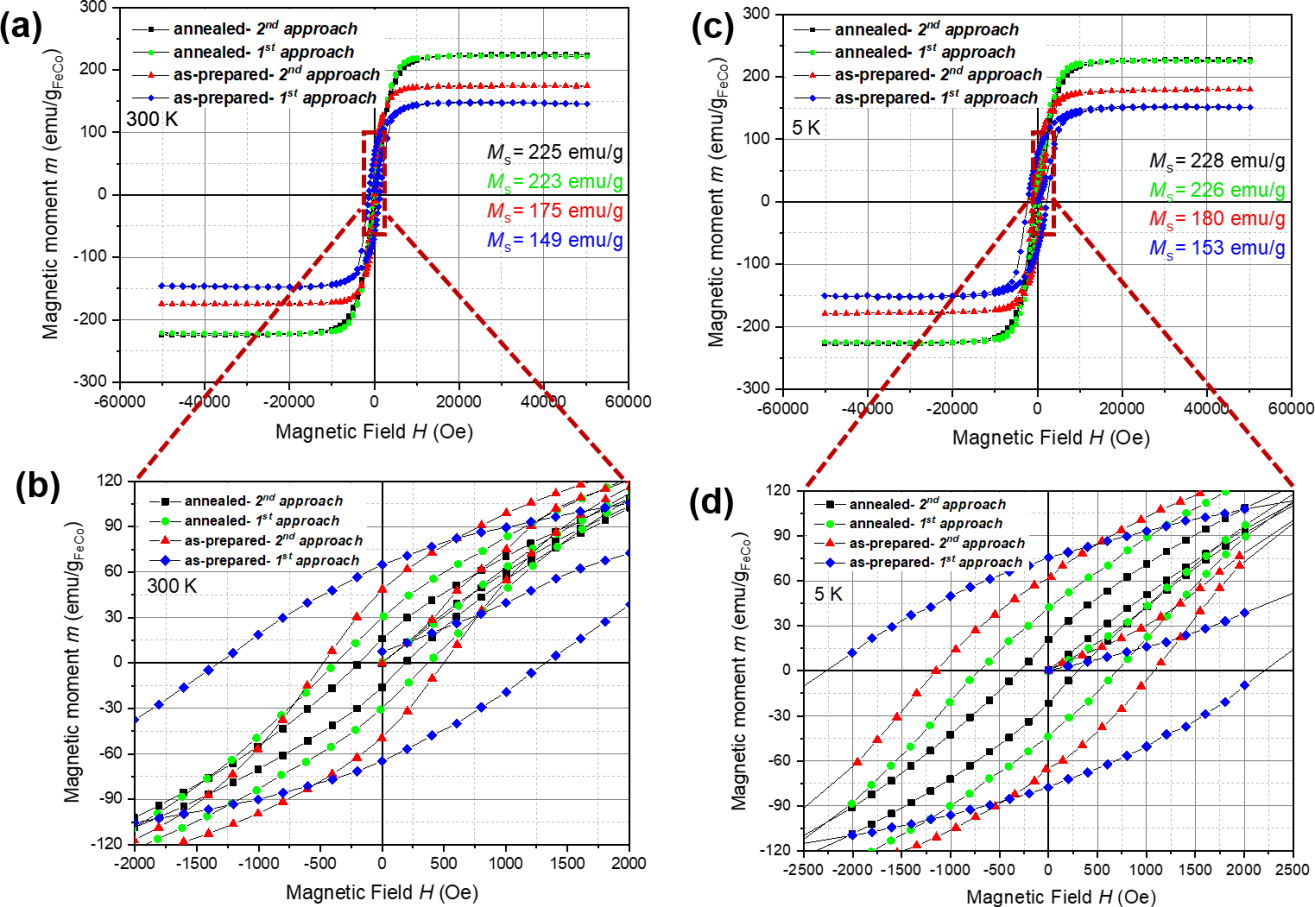
Hysteresis curves measured at a) 300 K and c) 5 K for the annealed and the as-prepared Fe_50_Co_50_@CNT material prepared by the first and second approaches. Data are normalized to the amount of magnetic material as obtained from the TGA measurements. Enlarged view of the hysteresis curves in b) 300 K and d) 5 K show the variation of the material hardness upon annealing.

The magnetic field dependence of the magnetization *M*(*H*) has been measured for the as-prepared and annealed samples of Fe_50_Co_50_@CNT prepared by both filling approaches as shown in Figure 7a and Figure 7c. Room temperature saturation magnetization *M*_s_ is shown in Figure 7a. The results obtained for the annealed samples are in a good agreement with those calculated from the Slater–Pauling curve for the same stoichiometry [50], and those also reported by Bardos [51] and Di Fabrizio et al. [52] in which the saturation magnetization at room temperature for equiatomic Fe–Co alloys prepared by arc melting equals to 233.5 emu/g. This finding implies that the relatively high saturation magnetization (known for the bulk material) is preserved even for the Fe_50_Co_50_ nanoparticles. Further, the magnetization data gives evidence of the importance of the annealing step, since the as-prepared samples exhibit a saturation magnetization significantly lower than the reported data for the bulk material of the same stoichiometry. This may be attributed to the lower crystallinity of the MNPs for the as-prepared samples compared to the annealed samples and to the formation of a bulk-like ferromagnetic core and a shell composed of disordered moments [32,34,53].

The temperature dependence of the saturation magnetization of the annealed samples prepared by both filling routes indicated a change of less than 2% when changing the temperature from 5 K to 300 K (Figure 7a,c). This small decrease in the magnetization upon heating to 300 K can be attributed to the high Curie temperature (*T*_c_) of the bulk Fe–Co alloys [20–21]. In other words, the saturation magnetization of the bulk Fe–Co alloys is preserved in the Fe–Co nanoparticles while we found that the Curie temperature is still well above 400 K.

Further information on the magnetism of the materials is demonstrated by the observed differences in coercivity, which is a size-dependent property appearing already in the size range studied here. The data in Figure 7 demonstrate a robust enhancement in the hardness of the magnetic nanoparticles of the annealed Fe_50_Co_50_@CNT samples compared to the bulk material. Coercive field (*H*_c_) measurements for the Fe_50_Co_50_ nanoparticles prepared by the first and second filling approaches at 300 K yield were *H*_c_ ≈ 373 ± 2 Oe and *H*_c_ ≈ 185 ± 2 Oe (Figure 7b), respectively. These values are approximately 200 times higher than for the bulk material at the same temperature (*H*_c_ (bulk) = 0.68 Oe) [22,54]. The increase in the coercive field as the particle size decreases can be attributed to the size dependence of coercivity in the vicinity of the critical size of domain formation in nanoparticles [55]. Briefly described, in this size regime, the coercivity of single domain (SD) magnets decreases upon size reduction while it increases in the multidomain (MD) state. The fact that the as-prepared samples have significantly larger coercivities compared to the annealed ones can hence be straightforwardly attributed to the small size of the MNPs stabilizing the SD state. On the other hand, the larger post-annealed MNPs are in the MD state and show smaller coercive fields.

It is worth mentioning that we were able to design different MNPs with different coercivities depending on the route of filling and heat treatment process. In other words, the as-prepared samples prepared by the first route have the highest hardness (*H*_c_ ≈ 1324 ± 4 Oe) compared to the as-prepared samples prepared by the second route (*H*_c_ ≈ 488 ± 18 Oe). This can be attributed to the fact that for samples prepared by the first approach, particles with a lower density occupy the hollow cavity of the CNTs due to the presence of H_2_O molecules (i.e., low filling yield). Hence, the effect of small particle size appears with minimum agglomeration. On the contrary, for samples prepared by the second approach, the high possibility of agglomeration takes place due to the presence of a higher number of particles (i.e., high filling yield) and thus larger particles are found. This explains the lower coercivity in comparison with samples prepared by the first filling approach. The same explanation applies for the annealed samples.

The symmetry in the shape of the hysteresis loops indicates the good stability of the prepared samples against oxidation. As known, these particles are subject to oxidation when they are exposed to air unless they are shielded by the carbon shell. To be specific, the presence of oxide layers would imply the presence of an antiferromagnetic shell around the ferromagnetic cores, i.e., the material would evolve the exchange bias effect where nanoparticles cooled under a magnetic field show a significant shift between the coercive field values at the positive (*H*_c+_) and the negative (*H*_c−_) sides [30,55–56]. For the prepared samples, and within the experimental error, equal values of *H*_c+_ and *H*_c−_ have been found, which is a further indication of the protective nature of the CNT shells. For clarity, a summary of all the data mentioned above are listed in Table 1 below.

**Table 1:** Physical properties of the magnetic nanoparticles under study.

Filling approach	Sample	*d*_TEM_	*d*_XRD_	TGA (wt %)	*M*_s_ (emu/g_Fe–Co_) (300 K)	*H*_c_ (Oe) (300 K)

1st approach	as-prepared	–	–	8 ± 1	149 ± 19	1324 ± 4
	annealed	–	–		223 ± 28	373 ± 2

2nd approach	as-prepared	16 ± 5	19 ± 4	20 ± 1	175 ± 9	488 ± 18
	annealed	58 ± 20	47 ± 1		225 ± 12	185 ± 2

## Conclusion

In this work, Fe_50_Co_50_ nanoparticles were successfully encapsulated within the hollow cavity of CNTs using two facile routes. Our study demonstrates that both wet chemical methods offer simple ways to fill CNTs with these nanoparticles in a well-defined manner without the need of vigorous conditions. Depending on the chosen procedure, we were able to influence the filling yield, size, magnetic moment, coercivity, as well as the appearance of the sample in terms of particles inside CNTs to those on the outer surface. Tuning several parameters, such as precursor concentration, CNT mass, sonication time, type and volume of washing agents, annealing temperature and time, resulted in filling of the CNTs with Fe_50_Co_50_ nanoparticles in a well-defined manner. These approaches can be extended to the filling of many other kinds of magnetic nanoparticles

The crystallinity of the Fe–Co nanoparticles was verified by powder XRD, HRTEM and nanobeam electron diffraction. No indication of oxide or carbide phases were detected, which means that the synthesis approaches guarantee CNTs as protective shells for the MNPs. The additional annealing step is mandatory for pure phase, highly crystalline Fe_50_Co_50_@CNT materials. The magnetic properties of the magnetic nanoparticles show preservation of the saturation magnetization at the nanoscale, whereas the hardness of the Fe_50_Co_50_@CNT sample is enhanced due to the size reduction, which could make them interesting for different applications. For example, industrial applications which require a high Curie temperature and coercivity (such as magnetic storage devices) and medical applications (where they are considered as good thermo-seed candidates for cancer thermotherapy) could benefit from this enhanced material.

## Supporting Information

File 1 EDX Measurements.
