# Supplementary material for: Single-crystalline FeCo nanoparticle-filled carbon nanotubes: synthesis, structural characterization and magnetic properties
Source: Beilstein J Nanotechnol. 2018 Mar 29;9:1024–34. doi: 10.3762/bjnano.9.95 (PMC5905243; doi:10.3762/bjnano.9.95)
Supplement: File 1 — EDX Measurements. [file Beilstein_J_Nanotechnol-09-1024-s001.pdf]

Supporting Information

for

Single-crystalline FeCo nanoparticle-filled carbon nanotubes: synthesis, structural characterization and magnetic properties

Rasha Ghunaim[*1,2,§], Maik Scholz[1], Christine Damm[1] , Bernd Rellinghaus[1], Rüdiger Klingeler[3,4], Bernd Büchner[1,5], Michael Mertig[2,6] and Silke Hampel[1]

Address: [1]Leibniz Institute for Solid State and Material Research Dresden, Helmholtzstrasse. 20, 01069 Dresden, Germany, [2]Institute for Physical Chemistry, Technische Universitaet Dresden, 01062 Dresden, Germany, [3]Kirchhoff Institute for Physics, Heidelberg University, Im Neuenheimer Feld 227, D-69120 Heidelberg, Germany, [4]Center for Advanced Materials (CAM), Heidelberg University, Im Neuenheimer Feld 225, D-69120 Heidelberg, Germany, [5]Institute for Solid State Physics, Technische Universitaet Dresden, 01062 Dresden, Germany and [6]Kurt-Schwabe-Institut für Mess- und Sensortechnik e.V. Meinsberg, 04736 Waldheim, Germany

Email: Rasha Ghunaim - r.ghunaim@ifw-dresden.de
* Corresponding author
[§] Tel: +49 (0) 351 4659 413

EDX Measurements

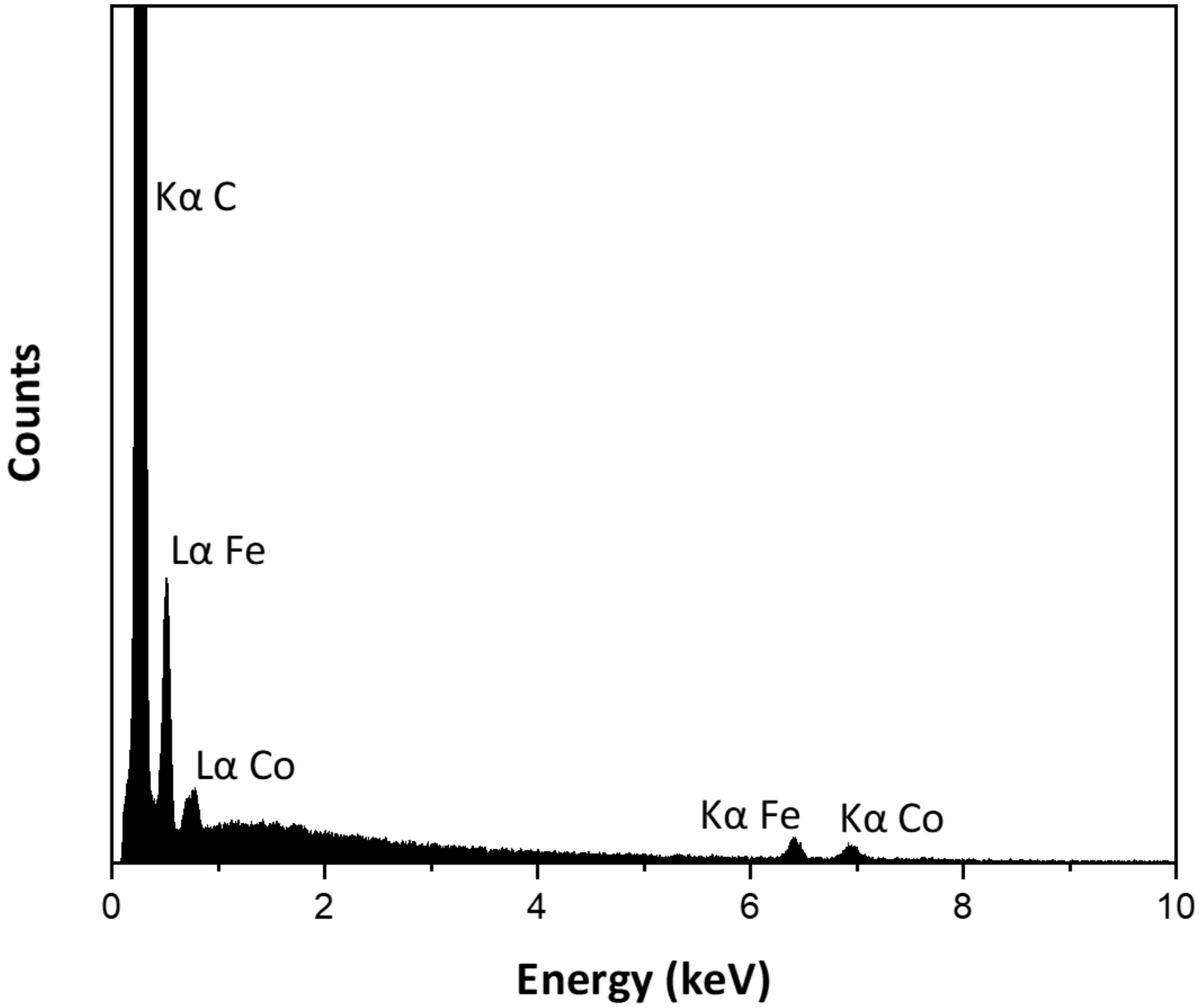

Figure S1: SEM-EDX quantitative measurement for the Fe/Co ratio over a selected area for an annealed sample of $Fe_{50}Co_{50}$@CNT, in which the ratio roughly corresponds to 1:1.
